# Epithelial Ovarian Cancer and the Occurrence of Skin Cancer in The Netherlands: Histological Type Connotations

**DOI:** 10.5402/2011/617082

**Published:** 2011-04-10

**Authors:** Catharina C. van Niekerk, Johan Bulten, André L. M. Verbeek

**Affiliations:** ^1^Department of Epidemiology, Biostatistics and HTA, Radboud University Nijmegen Medical Centre, P.O. Box 9101, 6500 HB Nijmegen, The Netherlands; ^2^Department of Pathology, Radboud University Nijmegen Medical Centre, P.O. Box 9101, 6500 HB Nijmegen, The Netherlands

## Abstract

*Background*. Patients with epithelial ovarian cancer have a high risk of (non-)melanoma skin cancer. The association between histological variants of primary ovarian cancer and skin cancer is poorly documented. *Objectives*. To further evaluate the risk of skin cancer based on the histology of the epithelial ovarian cancer. *Methods*. A cross-sectional study within a large population-based dataset. *Results*. Skin cancer was found in 2.7% (95% CI: 2.3–3.1) of the 5366 individuals forming our dataset. The odds ratio (OR) for endometrioid cancer in the ovary to skin cancer in the under 50 age group was 8.9 (95% CI: 3.2–25.0). The OR decreased in older patients to 1.2. *Conclusions*. Patients with epithelial ovarian malignancies show an increased risk of skin cancer. A significantly increased risk (4.3%) for endometrioid ovarian cancer was found in the group aged under 50.

## 1. Introduction

Epidemiological evidence suggests that individuals with skin cancers have a greater risk of developing other malignancies [1–6]. This is demonstrated for basal cell cancer, squamous cell cancer, and (non-)melanoma skin cancer. For postmenopausal women with nonmelanoma skin cancer, an odds ratio of 2.01 (95% CI: 1.61–2.50) was calculated for the association with all forms of ovarian epithelial cancers [6]. According to the sparse literature, which mainly consists of case series, the presence of primary histological ovarian carcinoma types and skin cancer is not well documented. More recently, Van Niekerk et al. [7] estimated excess risks of melanoma skin cancer (observed/expected ratio = 7.4) and non-melanoma skin cancer (basal cell cancer included; O/E = 1.6) for patients with epithelial ovarian cancer. This strong relationship prompted us to further evaluate the risk of skin cancer based on the histological type of epithelial ovarian cancer we have recently studied [8].

## 2. Materials and Methods

We examined the various histopathological types of epithelial ovarian cancer in relation to primary skin cancers using the Dutch nationwide pathology database (PALGA—Pathologisch Anatomisch Landelijk Geautomatiseerd Archief). Every record in the PALGA database contains a summary of the full pathology report and diagnostic codes similar to the Systematized Nomenclature of Medicine (SNOMED) classification of the College of American Pathologists. Because of the laborious nature of the review research, we used two random samples from PALGA.

From the first random sample of the years 1987–1993, we investigated 4577 patients. For the more recent years 1996–2003, we studied a smaller number of 789 cases to see whether the overall picture has changed. For these 5366 patients diagnosed with malignant or borderline malignant epithelial ovarian cancer, we also retrieved all the other histopathologically confirmed diagnoses of primary invasive malignancies of the skin diagnosed prior to, concurrent with, or after the ovarian tumour diagnosis. We reviewed both the diagnostic codes in the PALGA database and the corresponding pathology conclusions, that is, PALGA code and PALGA conclusion. This was also done for the skin malignant occurring neoplasms. Two experienced pathologists (G. P. V., J. Bulten) assessed all reports. Cases were excluded, if we were not sure that we were dealing with a metastasis of the primary malignant tumour of the ovary or a new secondary type of a tumour of the ovaries.

The Scientific Committee of PALGA approved the study protocol.

### 2.1. Data Analysis

First, we applied descriptive analysis to the PALGA records of patients having ovarian epithelial cancer and/or skin cancer. The association between histological type and the occurrence of skin cancer was evaluated by calculating the odds ratios. 

95% confidence intervals were calculated to address the precision of the estimated cancer rate and the relation between ovarian subtypes and skin cancer. The chi-square test was used to determine *P *values.

## 3. Results

In this study, 5366 cases of primary epithelial malignant ovarian cancer were selected from the PALGA registry, 4577 in the period 1987–1993 and 789 in the period 1996–2003. Of these 5366 patients, 145 cases of a second primary skin malignancy were reported. Analysis of the data from each period showed that disease occurrence between 1996 to 2003 did not differ from that of the larger database from 1987 to 1993. We therefore merged both datasets.

The 5366 individuals showed an occurrence rate of skin cancer of 2.7% (95% CI: 2.3–3.1); see Table 1. For the various subtypes of ovarian carcinoma, the rates for skin cancer were adenocarcinoma, 2.3% (95% CI: 1.5–3.1); clear cell carcinoma, 3.0% (95% CI: 0.8–5.2); endometrioid carcinoma, 4.3% (95% CI: 2.4–6.2); mucinous carcinoma, 2.5% (95% CI: 4–3.7); serous carcinoma, 3.0 (95% CI: 2.2–3.9); remainder types, 2.5% (95% CI 1.3–3.7). However, endometrioid ovarian carcinoma showed a significantly increased risk compared to the other types: *P* value =  .02. In Table 2, an odds ratio of 8.9 (95% CI: 3.2–25.0) was calculated for endometrioid carcinoma in the ovary in relation to skin cancer in patients younger than 50. In patients older than 50, the odds ratio decreased to 1.2.

## 4. Discussion

Our population-based study included all incident cases of basal cell carcinoma (104), melanoma (16), and other skin cancers (squamous cell carcinoma and Morbus Bowen, together 25) reported in the PALGA sample of 5366 ovarian cancer patients between 1987–1996 and 1996–2003. This study focused on the risk of skin cancer in ovarian cancer patients according to histological type as recently reported by Van Niekerk et al. [7], who calculated the observed versus expected risk for melanoma skin cancer to be 7.4 and for basal cell cancer 1.6.

The reasons for these increases are probably manyfold. In general, an association between skin cancer and a second primary cancer could reflect shared etiologic factors or biased ascertainment of new primaries as a result of increased surveillance [4, 9]. Immune suppression induced by therapy, exposure to ultraviolet radiation, and the primary disease could explain some cases. It should also be noted that individuals who are at high risk of skin cancer may have other risk-taking behaviours related to their increase in risk of a second primary cancer such as smoking, dietary intake, and physical activity levels [10]. The relationship with endometrioid ovarian carcinoma may however be more due to common genetic predisposition rather than secondary contributors such as chemotherapy and smoking.

In the sparse literature on ovarian cancer, and the occurrence of skin cancer most studies have been conducted in patients with a primary skin cancer and a new second primary ovarian cancer [1, 4, 6, 10–13]. Nugent et al. [11] concluded that females with a history of basal cell carcinoma were at greater risk of dying from ovarian cancer. Our results show that patients with epithelial ovarian malignancies show an increased risk for skin cancer, especially the group of patients with endometrioid carcinoma. Therefore, we suggest a careful surveillance of skin lesions in malignant epithelial ovarian cancer patients, in particular in patients with endometrioid ovarian carcinoma. Our study should stimulate new projects on shared carcinogenic pathways.

## Figures and Tables

**Table 1:** Patients with epithelial ovarian cancers according to histological type and the occurrence of skin cancer.

	Ovarian cancer	Basal cell carcinoma	Melanoma	Other skin cancer	Total skin cancer		
Histological type of ovarian cancer	*n*	*n*	*n*	*n*	*n*	%	95% CI^a^
Adenocarcinoma	1456	25	0	8	33	2.3	1.5–3.1
Clear cell carcinoma	236	6	0	1	7	3.0	0.8–5.2
Endometrioid carcinoma	460	12	4	4	20	4.3	2.4–6.2
Mucinous carcinoma^b^	733	10	5	3	18	2.5	1.4–3.7
Serous carcinoma	1640	38	4	7	49	3.0	2.2–3.9
Serous b.m.	161	1	0	0	1	0.6	0.0–7.0
Others^b, c^	680	12	3	2	17	2.5	1.3–3.7

Total	5366	104	16	25	145	2.7	2.3–3.1

^a^95% CI, confidence interval.

^b^Borderline malignancies included.

^c^Including mixed carcinoma, anaplastic carcinoma, malignant Brenner tumour, carcinosarcoma, adenosquamous carcinoma, squamous cell carcinoma.

**Table 2:** Odds ratio of endometrioid versus other ovarian carcinoma in relation to skin cancer and age at diagnosis of ovarian cancer.

	Skin cancer			Skin cancer		
	<50 yr			≥50 yr		
Ovarian cancer	total	yes	no	total	yes	no

Endometrioid carcinoma	122	7	115	338	13	325
All other histological types	1177	8	1169	3729	117	3612

	OR = 8.9 = (7 × 1169)/(8 × 115)			OR = 1.2 = (13 × 3612)/(117 × 325)		
	95% CI: (3.2–25.0)			95% CI: (0.7–2.2)		
	*X* ^2^ = 24.76; *P*-value = .0001			*X* ^2^ = .50; *P*-value = .24		
